# Beyond the eConsult platform: an implementation science perspective on tele-antimicrobial stewardship

**DOI:** 10.1017/ash.2026.10929

**Published:** 2026-10-14

**Authors:** Yasuaki Tagashira, Yoshiaki Gu

**Affiliations:** 1 Department of Infectious Diseases, Institute of Science Tokyo, Tokyo, Japan; 2 Center for Infectious Disease Education and Analysis (TCIDEA), Institute of Science Tokyo, Tokyo, Japan

To the Editor

We read with great interest the study by Itoh et al evaluating an electronic consultation (eConsult)-based tele-antimicrobial stewardship program using the ExpertTWIN system.^1^ The reduction in carbapenem use they report is encouraging. Consistent with the authors’ description of an integrated program, we suggest interpreting these findings primarily as support for a model that incorporates remote infectious diseases expertise into an existing stewardship workflow. The study does not isolate the contribution of the platform itself.

The intervention evaluated in this study was not the ExpertTWIN platform alone. Pharmacists identified patients receiving prolonged carbapenem therapy, off-site infectious diseases specialists reviewed individual cases and generated recommendations, the local antimicrobial stewardship team then vetted those recommendations, and pharmacists communicated them to treating physicians.^1^ ExpertTWIN provided the infrastructure through which this process occurred. The observed reduction therefore reflects this combined process rather than the platform in isolation. From an implementation science perspective, the platform’s contribution should be considered alongside specialist review, local case selection, and feedback delivery, consistent with implementation guidance emphasizing the integration of remote infectious diseases support with local personnel and organizational processes.^2^

We would also welcome the authors’ perspective on how the observed effect arose. Before implementation, the stewardship team—two non-infectious diseases physicians, two pharmacists, and one nurse—already conducted weekly postprescription review with feedback, and the number of feedback reports was similar in both periods (184 and 150).^1^ What was newly added was case-specific advice from infectious diseases specialists: of 214 recommendations, 135 (63.1%) were accepted.^1^ The case-selection criteria also changed between periods. Before implementation, the team reviewed designated agents used for ≥14 days; afterward, patients receiving carbapenems for ≥7 days were eligible, and from July 2025 those treated for 3–6 days were added as the weekly case target rose from 2–3 to 4–6 cases.^1^ Earlier and more intensive case identification is itself a stewardship intervention and could reduce carbapenem days of therapy independently of the platform or specialist input. This staged escalation also sits uneasily with a single-change-point model; reporting effects separately before and after July 2025 would help distinguish these influences. The mechanism underlying the observed reduction, however, remains uncertain. The acceptance of recommendations does not establish that specialist involvement increased the credibility of pharmacists’ advice, nor does it identify the contribution of any individual component. The platform’s structured documentation and asynchronous communication may also have facilitated delivery, but their independent effects were not evaluated. Specialist expertise might nonetheless be mobilized through other dependable channels, including scheduled telephone or video case conferences. For settings with limited infectious diseases resources, the practical question is what such a model requires locally.

Mean carbapenem use decreased from 5.2 to 3.8 days of therapy (DOT) per 100 patient-days at a 500-bed tertiary hospital with a substantial oncology caseload.^1^ By comparison, national surveillance data reported a median of approximately 1.8 DOT per 100 patient-days among Japanese acute-care hospitals during 2024–2026.^3^ Such comparisons, however, require attention to hospital size, case mix, reporting periods, and the composition of participating facilities; they cannot establish whether use at an individual hospital is excessive. Even so, preintervention use at the study hospital was appreciably above the national median, and the scope for reduction was correspondingly large. Baseline antimicrobial use and existing stewardship capacity may therefore modify the magnitude of benefit, although this study did not test effect modification. Furthermore, infectious diseases specialists judged a high proportion of reviewed cases to be inappropriate, although, as the authors note, this reflected targeted selection of prolonged carbapenem therapy rather than hospital-wide prescribing quality.^1^ Transportability to hospitals with lower baseline use or more established stewardship activities therefore warrants caution. Reduction in antimicrobial use is an important effectiveness outcome but does not necessarily indicate that an appropriate or optimal level of use has been achieved.

More broadly, this study illustrates the importance of applying implementation science principles to antimicrobial stewardship research. Langford et al recently emphasized the need to define the intervention clearly, consider implementation context and strategies, and evaluate both effectiveness and implementation outcomes when designing stewardship studies, a framework that this journal also recommends for antimicrobial stewardship intervention studies.^4^ Applied to remote stewardship, this framework suggests where the present report can be extended: the authors already report response preparation time, response time, and recommendation acceptance, providing useful information about delivery.^1^ Additional priorities are consultation uptake among eligible cases, fidelity to case-selection criteria, total specialist and local staff workload beyond response preparation, implementation costs including platform and personnel costs, and sustained use beyond the study period. These measures would complement antimicrobial-use outcomes and clarify the resources and workflow conditions required for adoption.^2,4^

Itoh et al provide important evidence that remote access to infectious diseases expertise can be incorporated into antimicrobial stewardship practice in a hospital without on-site specialists.^1^ The broader implication extends beyond any single eConsult system: such a model offers a route to making infectious diseases expertise accessible to hospitals that lack it, provided the local workflow and resources required to sustain it are in place. Because before–after designs bundle the modality of specialist input with the expertise it conveys, a cluster-randomized trial comparing asynchronous eConsult with an equivalent-intensity telephone or video alternative, measuring recommendation acceptance and fidelity alongside antimicrobial use, would be a valuable next step. Such an approach would help clarify not only whether remote stewardship is associated with improved antimicrobial use but also how and under what conditions it can be implemented successfully.

## Research transparency and reproducibility

No original patient-level data were generated for this Letter. Aggregate antimicrobial-use data were extracted from the Japan Surveillance for Infection Prevention and Healthcare Epidemiology database.
